# Blood Pressure Lowering May Decrease Cognitive Decline; But Are We Ready to Lower Blood Pressure in the Real World?

**DOI:** 10.33696/cardiology.2.014

**Published:** 2021

**Authors:** Madhuri Ramakrishnan, Gary Gronseth, Aditi Gupta

**Affiliations:** 1 Division of Nephrology and Hypertension, Department of Internal Medicine, University of Kansas Medical Center, Kansas City, Kansas, United States; 2 Department of Neurology, University of Kansas Medical Center, Kansas City, Kansas, United States; 3 Alzheimer’s Disease Center, University of Kansas Medical Center, Kansas City, Kansas, United States

Dementia and hypertension are highly prevalent, epidemiologically related chronic conditions disproportionately affecting older persons; approximately 97% of persons with dementia [1] and 66% with hypertension are over the age of 65 [2]. With increasing life expectancy, the prevalence of both dementia and hypertension is projected to increase further. Both conditions increase morbidity and mortality, and are among the largest contributors to healthcare expenditure – the total estimated expenditure on dementia in the United States in 2020 was $ 305 billion [3], and the annual expenditure on hypertension is estimated to be around $ 131 billion [4]. In addition, hypertension is an independent modifiable risk factor for dementia [5,6]. More recently, we analyzed data from randomized controlled trials (RCTs) to show that lowering blood pressure (BP) may slow decline in cognition [7]. Unfortunately, BP control has declined in the recent years, from 54% in 2013 to 44% in 2017 [8]. Here we will discuss some current logistical issues with hypertension management in older patients and future directions.

With up to half the cases of Alzheimer’s disease worldwide attributed to modifiable risk factors including hypertension, it would stand to reason that BP lowering would help with prevention and management of dementia. Indeed, it is estimated that BP lowering in midlife hypertension with 10% reduction in hypertension prevalence will reduce dementia by approximately 40,000 cases [9]. However, despite these data, BP lowering in older patients is fraught with complexities and challenges. First, the definition of hypertension and the target BP for older patients has changed over the years. The Joint National Commission (JNC)-7 guidelines for the treatment of hypertension published in 2003 recommended 140/90 mmHg as the threshold to initiate anti-hypertensive therapy, with a target of achieving a BP of <140/90 mmHg [10]. This was increased to 150/90 mmHg in 2014 under the JNC-8 guidelines in 2014 [11] and was mirrored by the American Academy of Family Physicians guidelines of 2017 [12]. After the landmark systolic blood pressure intervention (SPRINT) trial [13], the American College of Cardiology/American Heart Association (ACC/AHA) guidelines in 2017 lowered this threshold to 130/80 mmHg [14]. The 2018 European Society of Cardiology/European Society of Hypertension guidelines for hypertension management, published a year after the ACC/AHA guidelines however continued to recommend a threshold of 140/90 mm Hg for initiating treatment [15]. The latest Kidney Disease: Improving Global Outcomes (KDIGO) guidelines for hypertension recommend a target BP of <120/80 mmHg in adults with Chronic Kidney Disease (CKD) using unattended BP measurements [16], and over 35% of adults with CKD in the United States are over the age of 65 [17]. The trend over the years has been to lower BP goals. However, with a multitude of guidelines, each with a different BP goal, the message to lower BP is sometimes lost. Second, despite current evidence from RCTs and meta-analysis demonstrating safety and benefits of BP lowering in older patients [14], clinicians continue to have concerns about lowering BP in this population. Some of the concern is due to the fact that current evidence and hypertension guidelines are based on results of explanatory trials. These results may not be completely generalizable as explanatory trials have strict inclusion and exclusion criteria that tend to exclude sicker and frail patients such as those in nursing homes, and those with multiple comorbidities, orthostatic hypotension, severe kidney disease, history of stroke with residual deficits or balance issues [18]. While these patients are excluded from the trials, in the real world, clinicians still have to manage hypertension in these patients, and in the absence of pragmatic trials, clinicians often do not have enough evidence to guide management. In addition, while RCTs demonstrate overall safety, the aggregate data does not guarantee safety of an individual patient, which is still the responsibility of the physician.

There are also other barriers to achieving BP goals at the patient, provider, and health system level, some of which are listed in Table 1. In addition, there are clinical concerns about safety of tolerability of BP lowering and side effects of anti-hypertensive medications in select hypertensive older adults. One of such clinical concerns is that lowering BP can cause cerebral hypoperfusion and decline in cognition. We performed a systematic review and meta-analysis, to analyze the effect of BP lowering on cognitive decline in older adults [7]. This review was prompted by the clinical hesitancy to lower BP to current guidelines described above. We analyzed data from nine qualifying RCTs, including the Systolic Hypertension in the Elderly Program (SHEP) [19], the hypertension trial by the Medical Research Council [20], the Systolic Hypertension in Europe trial (Syst-Eur) [21], the Study on Cognition and Prognosis in the Elderly (SCOPE) [22], hypertension trial by Pantoni et al. [23], the Hypertension in the Very Elderly Trial-cognitive function assessment (HYVET-Cog) [24], The Telmisartan Randomized AssessmeNt Study in aCE iNtolerant subjects with cardiovascular Disease (TRANSCEND) [25], The Memory in Diabetes sub-study of the Action to Control Cardiovascular Risk in Diabetes study (ACCORD-MIND) [26], and SPRINT-MIND [27]. The PICO (population, intervention, control, and outcomes) criteria included RCTs on BP lowering with pharmacological agents in patients >60 years, with a pre-specified outcome of cognition, a standardized measure of cognition, presence of comparison group with either placebo, no intervention or a higher BP goal, and at least 1 year of follow up. We found a small improvement in cognition with BP lowering. Though the effect size for improvement in cognition is small, this study alleviates concerns for worsening of cognition with BP lowering. Furthermore, even a small positive effect is clinically significant due to the high prevalence of hypertension and dementia resulting in a large epidemiological impact. Indeed, the NNT to prevent mild cognitive impairment or probably dementia with this effect size was 63. We concluded that BP lowering did not cause worsening of cognitive decline in the older population. Consistent with our results, [7] other recent meta-analyses also indicate that lowering BP does not worsen cognitive decline. [28,29].

Some older studies have linked treatment of hypertension with cerebral hypoperfusion [30,31]. While there is a U-shaped association between systolic BP and cognition [32,33], prospective observational studies have demonstrated that BP lowering reduces risk of dementia [34]. Other recent studies, including some RCTs are also in agreement that lowering BP does not decrease cerebral perfusion- this might be due to cerebral autoregulation [35,36]. In a recent RCT, intensive lowering of systolic BP (mean of less than 130 mmHg) did not reduce cerebral perfusion even in participants with severe small vessel cerebral disease [37].

BP lowering is beneficial. However, consideration has be given to potential side effects and tolerability of anti-hypertensive therapy which can lead to non-adherence - the most common cause of failure to achieve BP goals. Patients on anti-hypertensive medications should be asked about symptoms of low BP or side effects of medications such as lightheadedness, syncope, falls, increased fatigue, drowsiness, orthostatic hypotension, cough and sexual dysfunction, and kidney function and electrolytes need to be monitored closely. In addition, polypharmacy and drug interaction also need to be considered. Combination pills should be encouraged to decreased pill burden and increase medication adherence.

In summary, despite a plethora of clinical trials and strong evidence to support BP lowering, we have not been successful in achieving BP goals in our clinics. Hypertension remains vastly undertreated, with only 30% of patients over 65 years achieving a BP of <140/90 mmHg [38]. Real world management of hypertension has added difficulties when compared to BP lowering in privileged clinical trial setting with financial and personnel assistance. It is important to understand that methods used in clinical trials may not be directly transferable to clinical care in real world clinical practice. Barriers to achieving BP goals need to be evaluated and addressed (Table 1). Perhaps we need to revisit our current strategies for hypertension management and implement effective practices such as home BP monitoring, use modern technology and team science for remote BP monitoring and management. We also need to find practical solutions to barriers such as inability to obtain accurate BP measurement in clinics. We need better strategies to improve patient education, engagement and empowerment. It is time to move to pragmatic trials of BP lowering in the real world.

## Figures and Tables

**Table 1:** Barriers to achieving goal blood pressure.

Patient level	Lack of knowledge [5,39]
	Inaccurately measured BP, use of inaccurate or non-validated BP cuff
	Adherence to medications [40,41] and home BP monitoring
	Logistical issues: access to care, distance from clinic, lack of time for clinic visits and transportation
Provider level	Lack of clinical decision support or treatment algorithms, clinical inertia, lack of physician incentives to lower BP [42–46]
	Inability to set accurate BP goals due to incorrect in-clinic BP [47], masked hypertension or white coat hypertension
	Limited time and resources for patient counselling in an already busy clinic
Health system level	Poorly aligned incentives, lack of decision support tools or their implementation, and lack of feedback [48–51]
	Lack of clinic space and resources for timely follow up for hypertension management [42–46,52]
	Improper and inconsistent BP measuring techniques in clinics [47]
